# Clinical Laboratory Experience With Prenatal cfDNA Screening in Triplet Pregnancies

**DOI:** 10.1002/pd.6812

**Published:** 2025-05-08

**Authors:** Erica Soster, Brittany Dyr, Samantha Caldwell, Savannah Moore, Eyad Almasri, Hany Magharyous, Juan‐Sebastian Saldivar, Phillip Cacheris

**Affiliations:** ^1^ Labcorp San Diego California USA; ^2^ Wake Forest University Winston‐Salem North Carolina USA

## Abstract

**Objective:**

Prenatal cfDNA screening is the most sensitive and specific screen for common aneuploidies in singleton and twin pregnancies and has been endorsed by several professional societies as a first‐tier screen or contingent screen. However, data for triplet pregnancies is lacking, as these pregnancies are relatively uncommon and obtaining sufficient data for a robust analysis of test performance is challenging.

**Method:**

This study presents a retrospective review of over 1500 samples from triplet pregnancies screened via cfDNA for common aneuploidies.

**Results:**

Mean patient age was 34 years, while mean gestational age was 13 weeks. The most common indication for testing was patient age, representing > 60% of the cohort. There were 13 screen‐positive cases (1.01%), 270 NR cases (17.32%), and the remainder were screen‐negative. Complete or partial genetic and/or obstetric outcome information (including birth and neonatal outcomes) was available for 147 samples, including all 13 positive cfDNA samples. No false positive or false negative cases were identified.

**Conclusion:**

The data from this study support the notion that cfDNA screening in triplet pregnancies is a reasonable approach given the lack of alternative screening options for these patients and that the performance likely approaches that of twin pregnancies, albeit with a higher no‐call rate.

**Summary**

- What's already known about this topic?
  ◦ Prenatal cfDNA screening is the most sensitive and specific screen for common trisomies in singleton and twin pregnancies.
  ◦ Data on cfDNA in triplet pregnancies is lacking.
- What does this study add?
  ◦ This is the largest cohort of samples (> 1500) from 1472 unique triplet pregnancies with cfDNA screening, including 13 positive cases.
  ◦ Complete or partial genetic/obstetric outcome is available for 147 cases (from 141 unique pregnancies), including all screen‐positive cases. No false positive or false negative cases were identified.

## 1 Introduction

Prenatal cell free DNA (cfDNA) screening (also known as non‐invasive prenatal testing, or NIPT) has been widely adopted for screening for fetal aneuploidy in singleton pregnancies, and to a lesser extent in twin pregnancies. However, the issue of whether prenatal cfDNA screening is a reasonable approach in triplet pregnancies is still in question. A handful of triplet pregnancies were included in the initial laboratory validation of a prenatal cfDNA screening assay in multifetal pregnancies, although the study was predominantly twins [1].

Triplet pregnancies present several challenges for clinicians, not the least of which is screening and diagnosis of prenatal aneuploidy. Triplet pregnancies have increased risks for several adverse outcomes [2, 3]. In some cases, fetal aneuploidy status may be an informative piece of data when deciding on procedures such as multifetal pregnancy reduction or selective reduction. However, there have historically been no good options for screening triplet pregnancies for aneuploidy [4]. While NT screening may be performed via ultrasound, it may be technically challenging to obtain adequate images of all three fetuses due to fetal position and/or patient body habitus. The same limitations also apply to second trimester anatomy scans at 18–20 weeks [2]. There are no data on the performance of biochemical serum screening options in triplet pregnancies [3]. Even genetic testing such as chorionic villus sampling or amniocentesis may be more technically difficult in triplet pregnancies, especially if a sample is needed or desired from all three fetuses, as this may require three separate needle insertions [3]. Procedure‐related risks may be higher in multifetal pregnancies as compared to singletons [3, 5].

On the other hand, triplet pregnancies are relatively uncommon and aneuploidy in triplet pregnancies is even less common. Thus, obtaining enough data to provide a robust analysis of any screening assay for aneuploidy in triplet pregnancies is a challenge. The International Society for Prenatal Diagnosis (ISPD) eloquently summarizes this, extrapolating that nearly 11 million total population cases of prenatal cfDNA screening would be needed to identify 10 affected cases of trisomy 21 in roughly 3400 triplet pregnancies (assuming a similar first trimester prevalence as for singletons of 1:340) [6]. The statement goes on to say: “Based on our current knowledge of how cfDNA testing distinguishes the underlying genetics between mother and fetus, one could extrapolate this knowledge from screening singletons and twins to a theoretical performance in triplet pregnancies… At least for shotgun methods, the screening performance should approach that found for twin pregnancies [6].” However, they also acknowledge that non‐reportable (NR) results owing to insufficient fetal fraction will likely result in higher test failure rates.

Because many commercial cfDNA laboratories do not accept triplet samples, previous studies involving triplet pregnancies with prenatal cfDNA screening are limited, but the data is promising. Recently, Zakaria et al. explored the performance of cfDNA in triplet pregnancies as a first‐tier test in over 200 pregnancies. The study identified three positive trisomy 21 cases and one trisomy 18 case, with a 2.4% NR rate [7], no false negative results and only one false positive. Two other studies included several triplet pregnancies but did not identify any positive cases [8, 9].

A previous study from our laboratory focused primarily on twin pregnancies, but did include over 700 triplet pregnancies, with limited follow‐up [10]. Those triplet samples are included in this cohort.

In this retrospective analysis, over 1500 samples from triplet pregnancies were identified. This study aims to explore the demographics and outcomes of those cases to add to the existing, although limited, literature on the use of cfDNA in triplet pregnancies. To our knowledge, this is the largest cohort of triplet pregnancies with cfDNA in the literature.

## 2 Methods

A retrospective analysis was performed on a cohort of triplet pregnancies with clinical prenatal cfDNA screening at a single commercial laboratory (Labcorp) beginning with multifetal assay validation in 2012 through October 2022. This cohort was derived by searching for samples that were accessioned from a triplet pregnancy on the test requisition form (TRF) among over three million prenatal cfDNA screening samples. Whole blood was collected using cfDNA collection tubes (Streck, Omaha, NE) and shipped to the laboratory for testing. Blood samples were subjected to DNA extraction, library preparation, and genome‐wide massively parallel sequencing as previously described [11], in a commercial laboratory certified by the Clinical Laboratory Improvement Amendments (CLIA) and accredited by the College of American Pathologists (CAP). Briefly, cfDNA fragments were subjected to genome‐wide sequencing and unique reads were mapped to 50 kilobase (kb) bins, normalized, and counted. For analysis of the common trisomies, chromosomes 21, 18, and 13 are assessed for a contiguous over‐representation of DNA along the entire chromosome. In triplet and higher‐order multifetal pregnancies, the assay will report on the presence or absence of the Y chromosome but does not predict the number of male fetuses when the Y chromosome is detected.

In multifetal pregnancies, sample‐specific fetal fraction requirements are adjusted in proportion to the number of fetuses, with consideration to the sequencing noise of the sample, using a metric deemed “signal to noise ratio,” or SNR as of September 2016 [12] (prior to SNR, a static fetal fraction threshold of three times the singleton fetal fraction [3%–4% depending on the assay version] was utilized). However, the methods to estimate fetal fraction and fetal fraction/SNR thresholds have evolved as the assay has been continuously improved over time [13, 14]. The implementation of SNR allowed for consideration of both the fetal fraction (signal) and the sequencing noise to determine whether an individual sample was reportable. In short, this allows for samples to be reported at lower fetal fractions if the sequencing data is of sufficient quality. Therefore, a sample must meet both a minimum fetal fraction requirement (at least 2.5% for singletons) and an SNR requirement to be reported. In general, the SNR requirement for triplets is three times that of singleton pregnancies, to allow sufficient representation from the entire pregnancy in the sample. Non‐reportable (NR) samples may fall into one of two different groups. Samples without sufficient fetal fraction (and/or failing to meet SNR in later versions of the assay) resulted as “quantity not sufficient” (QNS), while samples failing to meet other laboratory quality metrics resulted as a technical non‐reportable (TNR). Post‐test genetic counseling was available to any patient with a positive or non‐reportable (NR) cfDNA screening result.

Patient demographics and indications for testing were recorded as provided on the TRF completed by the ordering clinicians; not all fields are required for testing. Gestational age (GA) and patient age were entered as whole numbers, rounded to the nearest whole week or the nearest whole year. Patient weight is not required and was not available for all specimens. Turnaround time (TAT) is defined as the number of in‐lab calendar days beginning when the specimen is received at the laboratory until the release of results.

Indication for testing is also not a required field on the TRF. Of note, for the study cohort, samples that were indicated by the provider as advanced pregnant patient age were maintained as such. For samples with no provided indication for testing, if patient age was 35 or older, those cases were assigned to the advanced patient age group as well. Although some providers may use a different cutoff for pregnant patient age in the context of a multifetal pregnancy, to be conservative, 35 was maintained as the “advanced age” utilized in reassigning cases. All other samples with no indication provided (and patient age < 35), were grouped as no high‐risk indication for aneuploidy provided. Some cases were indicated as abnormal serum biochemical screening by the ordering provider; although traditional serum screening is not typically available for triplet pregnancies, there was inadequate information provided for those cases to determine the rationale for that indication, so they were left as serum screening.

Collection of outcomes was approved under AspireIRB under the clinical protocol SCMM‐RND‐402. Clinical and diagnostic genetic testing outcomes were obtained from three different sources. First, ad hoc feedback is routinely collected and documented in the clinical laboratory database by laboratory genetic counselors and genetic counseling assistants. Second, cfDNA samples were cross‐referenced with genetic testing results (FISH, karyotype, and microarray) submitted to Labcorp from chorionic villus, amniocentesis, cord blood or neonatal peripheral blood, and products of conception specimens during the study timeframe. Third, genetic counselors and genetic counseling assistants contacted the ordering provider on all triplet cfDNA samples accessioned from 2020 through October 2022 (positive, negative, or non‐reportable) to try to obtain as many outcomes as possible.

Samples were considered a “true positive” if at least one affected fetus was confirmed by diagnostic genetic testing following a screen‐positive cfDNA result. Samples were considered a “true negative” if diagnostic genetic testing results were reported in a case that was screen negative by cfDNA. If genetic testing on the fetuses or neonates was not available, birth outcomes noting normal neonates were also accepted. Specifically, in the context of triplet pregnancies, this meant that the providers reported no phenotypes consistent with the common trisomies during pregnancy or at birth based on neonatal assessment. Isolated IUGR and/or preterm birth or a fetal demise were not counted as a phenotype as this can be relatively common in triplet pregnancies in the absence of aneuploidy [2, 3]. A “likely negative” was defined as a negative cfDNA result in a case with no additional genetic testing along with normal ultrasounds late in gestation but no birth outcomes confirming an absence of phenotype. Some cases (positive, negative, and no‐result cfDNA samples) had available outcome information but could not be clearly assigned to one of the above categories and were designated as “other”. These cases typically had some outcome information available, such as a fetal demise, growth restriction, or a pregnancy reduction, but without enough information to designate the case as true (or likely) negative or true positive. False negatives were defined as cases in which genetic testing confirmed at least one affected fetus with a negative cfDNA sample. Meanwhile, false positives were defined as cases in which genetic testing and/or birth outcomes showed three unaffected fetuses.

For fetal sex concordance, if the cfDNA assay did not detect Y chromosome material, genetic and/or phenotype confirmation of female fetal sex for all three fetuses was required. For cases in which Y chromosome material was detected by the cfDNA assay, diagnostic genetic testing and/or phenotype confirmation of at least one male fetus was sufficient to determine concordance. A discordant fetal sex case was defined as a case in which the assay did not detect Y chromosome and at least one fetus was confirmed as male by diagnostic genetic testing and/or phenotype, or a case which did detect Y chromosome and genetic and/or phenotype information for all three fetuses showed they were all female.

Study data were described using rates, counts, and measures of central tendency. Comparisons between cohorts were performed using Wilcoxon rank sum tests with continuity corrections using R 4.2.2 and the packages dplyr, and plyr. A *p* value of < 0.05 was considered statistically significant. Positivity rates were calculated based on reportable samples.

## 3 Results

### 3.1 Cohort Overview and Demographics

**FIGURE 1:**
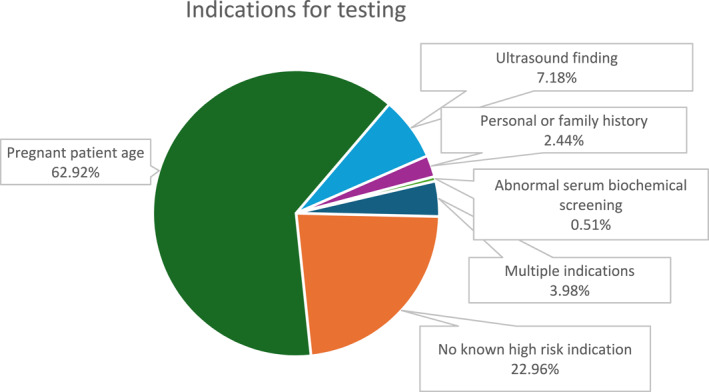
Indications for testing in the study cohort.

A total of 1559 samples from 1472 unique triplet pregnancies were sent for cfDNA screening during the study period. The average pregnant patient age in the cohort was 34 years (median 35 years, range 18–49 years) and the average gestational age at cfDNA screening was 13 weeks (median 12 weeks, range 9–40 weeks). The average patient weight was 76.19 kg (median 72.57 kg, range 39.46–195.05 kg). Specific gestational age was available for ∼98% of samples, while patient weight was available for ∼90% of samples. Figure 1 shows the indications for testing, with patient age being the most frequent. The mean TAT was 4.93 days.

### 3.2 Laboratory Metrics

**FIGURE 2:**
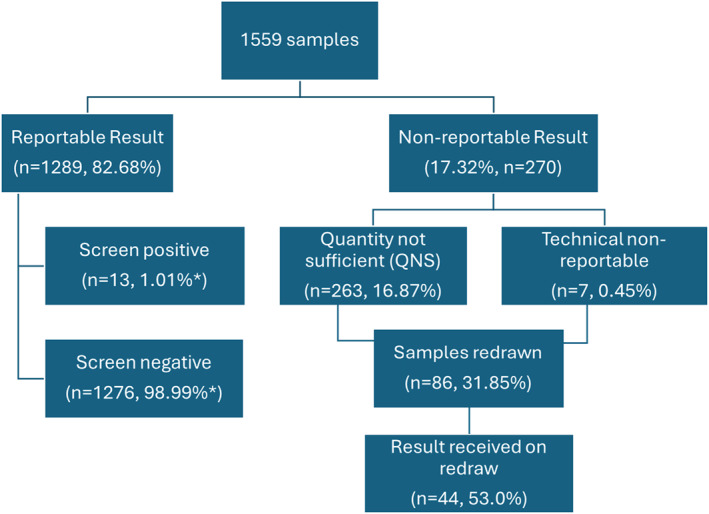
Flowchart of the samples in the study cohort. * Screen positive and screen negative rates are calculated based on reportable samples.

Most samples received a screen negative result (81.85% of total cohort, 98.99% of reportable cohort, *n* = 1276), while a total of 270 did not receive a reportable result (NR, 17.32%) comprised of 263 (16.87%) with a “quantity not sufficient” result (QNS) and 7 (0.45%) with a technical non‐reportable (TNR). There were 13 samples (from 13 unique pregnancies) with a positive screening result (0.83% of total cohort, 1.01% of reportable cohort) comprised of 11 positive trisomy 21 samples, 1 positive T18 sample, and 1 sample that flagged positive for trisomy 18, but upon further review, was flagging due to a partial duplication on chromosome 18. This was reported as a duplication of 18p11.32‐p11.21 of approximately 14.8 megabases. Of note, this region would not include the typical FISH probe for chromosome 18 that hybridizes to the centromere. There were no screen‐positive cases of trisomy 13 in the cohort. Details for the positive cases are summarized in Table 1, while Figure 2 shows a summary flowchart of the samples in the study cohort.

**TABLE 1:** Summary of clinical details and outcome information for the screen‐positive cases.

Case ID	cfDNA result	Pt age	GA (wks)	Indication	Fetal fraction	Concordance	Outcome notes
1	Positive—Trisomy 21; + Y chr	35 y	10	Patient age	25.24%	TP	CVS for fetus B and C was 46,XY; amniocentesis for fetus A was positive for trisomy 21.
2	Positive—Trisomy 21; + Y chr	42 y	12	USF, patient age	18.20%	TP	Fetus B positive for trisomy 21 on amniocentesis; selective reduction to twins.
3	Positive—Trisomy 21; + Y chr	42 y	11	Patient age	11.57%	TP	Fetus B positive for trisomy 21 on diagnostic testing (unspecified); selective reduction with uncomplicated delivery of twins at 38 weeks.
4	Positive—Trisomy 18; + Y chr	43 y	10	USF, patient age	11.73%	TP	Demise of one triplet (after cfDNA); amniocentesis on surviving twins showed one fetus with trisomy 18 and one unaffected fetus.
5	Positive—Trisomy 21; No Y chr	42 y	9	Patient age	9.36%	TP	IVF conception; trichorionic, triamniotic; at time of CVS, triplet A had increased NT, reduced without diagnostic testing.
							Ultrasound later in pregnancy showed EIF and growth lag of (now) twin A; delivery at 35 weeks. Diagnosed after birth with VSD and down syndrome by microarray.
6	Positive—Trisomy 21; + Y chr	35 y	17	Patient age	13.05%	TP	IUI conception, no reported ultrasound findings. Fetus A and fetus C unaffected by karyotype and microarray on unspecified sample type. Fetus B with confirmed trisomy 21.
7	Positive—Trisomy 21; No Y chr	38 y	11	Patient age	7.04%	Other	No diagnostic testing. Healthy livebirth of two babies, other fetus demised (timing unspecified)
8	Positive—Trisomy 21; + Y chr	39 y	9	Patient age	11.08%	TP	Amniocentesis confirmed trisomy 21 in fetus A; fetus B and C unaffected.
9	Positive—chr 18 duplication; + Y chr	35 y	18	Patient age	15.42%	Other	cfDNA data suggestive of duplication of 18p11.32‐p11.21; relayed to the provider.
							One fetus with growth restriction. No additional outcome information.
49	Positive—Trisomy 21; + Y chr	41 y	11	Patient age	16.78%	TP	Fetus A with confirmed trisomy 21 on amniocentesis microarray; fetus B and C unaffected.
101	Positive—Trisomy 21; + Y chr	35 y	10	USF, patient age	9.25%	TP	Amniocentesis on fetus with cystic hygroma confirmed trisomy 21. Selective reduction of that fetus. The remaining two fetuses had no testing and were delivered with no concern for trisomy 21.
119	Positive—Trisomy 21; + Y chr	38 y	20	Patient age	20.66%	TP	Diagnostic testing (unspecified) showed one fetus with trisomy 21 and remaining two fetuses unaffected.
143	Positive—Trisomy 21: + Y chr	40 y	13	Patient age	11.34%	TP	Amniocentesis showed one fetus with trisomy 21 and remaining two fetuses unaffected.

Abbreviations: + Y Chr = Y chromosome detected in sample, cfDNA = prenatal cfDNA screening, chr = chromosome, CVS = chorionic villus samples, EIF = echogenic intracardiac focus, GA = Gestational age, IUI = intrauterine insemination, IVF = in vitro fertilization, No Y chr = No Y chromosome detected in sample, NT = nuchal translucency, Pt = patient, TP = true positive, USF = ultrasound finding(s), VSD = ventricular septal defect, Wks = weeks, y = years.

The average fetal fraction of the overall cohort was 11.79%. Of the 270 samples with no result, there were 85 samples that were redrawn (31.12% of the NR cohort). Of those 85, the majority (*n* = 82) were initially QNS and 3 were initially TNR samples. For the redrawn samples, a result was received roughly half the time (53.7%, *n* = 44). The remaining samples did not receive a result on the redrawn specimen; in all cases, this was due to low fetal fraction (QNS) on the redrawn specimen. For samples with redraws, the average gestational age change between samples was 2.73 weeks and the average fetal fraction change was 1.50% (as noted in the methods, the gestational age is recorded in the laboratory information system rounded to the nearest whole week). The average gestational age of the initial specimens was 12.22 weeks with an average fetal fraction of 7.81%, whereas the average gestational age of the subsequent specimen was 14.93 weeks, and the average fetal fraction was 9.31%. Most redrawn specimens were collected within 3 weeks of the initial specimen.

A statistical comparison of the demographics between the QNS/TNR cohort and the reportable cohort showed that there was no significant difference in gestational age (*p* = 0.2634) or patient age (*p* = 0.5239). However, there were significant differences in the patient weights (*p* < 2.2e‐16). Supporting Information S1: Table S4 shows the median, mean, and IQR for weights, fetal fractions, gestational ages, and patient ages of the two cohorts.

### 3.3 Outcomes

There were 147 (9.43%) samples (from 141 unique pregnancies) that had at least partial diagnostic genetic testing and/or clinical outcomes available. These results are summarized in Table 2. Of note, there were no confirmed false positive or false negative cases in the cohort and all 13 of the screen‐positive cases had at least partial outcome information available. The outcome details for these 13 cases are also included in Table 1. Eleven of the 13 screen‐positive cases were confirmed on diagnostic testing, while 2 samples had “other” outcomes. One was a positive trisomy 21 sample with the demise of one fetus and healthy livebirths noted for the other two babies. Meanwhile, the 18p duplication case reported growth restriction for one fetus. For the screen‐negative samples, there were 94 samples that were unaffected by genetic testing and/or newborn outcomes. There were 29 samples with “other” outcomes and negative cfDNA screening results for which concordance could not be determined. Additionally, although limited in outcomes, there were no affected cases identified in the NR samples with available outcome information (*n* = 11, 4.07% of NR samples, 1 case with genetic testing, and 10 cases with obstetric outcomes that did not have any overt signs of aneuploidy). Of note, there were five pregnancies in the outcomes cohort that had initial samples which were QNS but were negative on repeat sampling and one pregnancy with two QNS samples. Details of samples with available outcome information are available in Table 1 for screen positive samples, and Supporting Information S1: Tables S1 and S2 for non‐reportable and screen negative samples, respectively.

**TABLE 2:** Concordance of cfDNA results and diagnostic testing and pregnancy/neonatal outcomes for 147 cases with available outcomes.

	Affected on diagnostic testing	Unaffected by diagnostic testing or newborn outcomes	Other (concordance cannot be determined)
Positive cfDNA result	11 (10 T21, 1 T18)	0	2^a^
Negative cfDNA result	0	94	29
Non‐reportable cfDNA result	0	11^b^	NA

^a^ One case (#7) with fetal demise and two healthy neonates and 1 case (#9) with IUGR.

^b^ For full details of cases, please see Supporting Information S1: Tables S1 and S2.

Furthermore, regarding fetal sex (presence or absence of Y chromosome), sufficient outcome information was available for 98 samples (77 samples with Y chromosome detected, and 21 samples with no Y chromosome detected). All but one sample was concordant for Y chromosome presence/absence; the one discordant case was a sample in which the assay detected the Y chromosome, but all three fetuses were female. That case was drawn at 9 weeks gestation and had a known demise at 6 weeks of the fourth fetus. Review of the sequencing data for this case showed that while the Y chromosome was detected, the Y chromosome contribution was at a lower level than expected and could potentially be derived from the demised fetus. Based on these 98 samples, the concordance rate for the presence/absence of the Y chromosome was 98.98%. Details of cases with available fetal sex outcomes are available in Supporting Information S1: Table S3.

### 3.4 Higher Order Multifetal Pregnancies

A small subset of cases (*n* = 26) was identified with prenatal cfDNA screening and more than three living fetuses. Although not included in the main analysis of the study, it is worth noting that cfDNA screening for these pregnancies is technically feasible, although limited by the increasing sample‐specific fetal fraction requirements with the increasing number of fetuses. This is reflected by the 42.3% (*n* = 11) QNS rate in these cases. The remaining 15 cases were all screen negative and there were no genetic or clinical outcomes available for any of the 26 cases, so assessment of test performance is not possible. The indications for testing were split between patient age (*n* = 14) and no known high‐risk indication/no indication provided (*n* = 12). The average patient age was 33.5 years and the average gestational age (based on 23 cases with GA provided) was 14 weeks, while the mean fetal fraction was 9.91%. There were 18 cases that indicated four fetuses, 5 cases that indicated five fetuses, and 1 case each indicating six, eight, and nine fetuses. All 15 reportable cases (meeting the fetal fraction requirements) had either four or five fetuses; none of the other higher‐order multifetal cases reached a sufficient fetal fraction to be reported.

## 4 Discussion

Most professional society recommendations on aneuploidy screening do not address triplet pregnancies or recommend against it, as summarized in the ISPD guidelines [6]. Consistent with previous literature, this cohort shows promising performance of prenatal cfDNA screening in triplet pregnancies, especially considering the absence of other options [7, 10]. Although the outcome information in this cohort is limited, all positive samples with complete outcome information were confirmed and no false negatives were identified. There was insufficient outcome data to calculate a test sensitivity, specificity, PPV, or NPV for this cohort. Fetal sex prediction via the presence or absence of Y chromosome material appears to be relatively reliable and consistent with performance for singleton and twin pregnancies [15, 16, 17]. Classification of outcomes in this cohort was particularly challenging, given the intricacies of triplet pregnancies and the increased risk of obstetrical complications. Irrespective of aneuploidy status, these pregnancies have a significant risk of miscarriage, fetal or neonatal demise or stillbirth, hypertensive disorders, and increased risk of preterm or very preterm delivery [2, 3]. Because of this, many cases were unable to be classified into concordant or discordant categories, given the small possibility that some of those same complications could also be associated with aneuploidy.

The NR rate in this study was slightly lower than that reported in an overlapping cohort from the same laboratory, likely attributable to assay improvements over time [10]. The NR rate in the other large triplet cfDNA cohort was significantly lower; that laboratory does not use a cut‐off for low fetal fraction [7]. Most of the non‐reportable samples in the present study were QNS, indicating that they failed to meet the fetal fraction requirements.

The strength of this study is that it is the largest cohort currently available with demographic and laboratory data on triplet pregnancies undergoing prenatal cfDNA screening. This cohort also has the most affected samples currently in the literature, with diagnostic genetic testing confirming most of those cases. However, one of the major limitations of this study is the overall lack of outcomes, particularly in screen‐negative and NR samples. Although no false negatives were reported to the laboratory by providers, we cannot say with certainty whether some false negatives went unreported. Furthermore, most of the affected cases were trisomy 21; the data around T18 remains limited and there were no cases of T13. This may be in part due to the frequency of ultrasound findings associated with those aneuploidies in the first trimester that may influence a patient to pursue additional genetic testing or other reproductive options in the context of a triplet pregnancy. Zakaria et al. had more outcomes overall and a higher percentage of outcomes but had a smaller overall cohort and fewer affected cases [7].

Understanding the expected incidence of trisomies in triplet pregnancies is complex. Just as the risk for aneuploidy is not “doubled” in dizygotic twin pregnancies, one cannot expect it to be “tripled” in triplet pregnancies arising from three conceptions, not to mention the various permutations of zygosity within triplet pregnancies. Thus, it is challenging to compare the incidence of aneuploidy in this cohort with a reference. If all cases of screen‐positive results are presumed to be affected, the incidence of aneuploidy in this cohort is 13/1559 (0.83%), or ∼1 in 120. However, given that one of the positive cases appears to be a CNV on chromosome 18, the incidence could also be calculated as 12/1559 (0.77%), or ∼1 in 130. The American College of Obstetricians and Gynecologists suggests a second‐trimester risk for a trisomy of 21, 18, or 13 as ∼47 in 10,000, or ∼1 in 213 for pregnant patients at age 35 [4]. The positivity rate in this cohort is higher than this risk value. Even if only the confirmed cases are considered, the cohort incidence is ∼1 in 140. This may be due to a selection bias of which cases were sent for prenatal cfDNA screening on the part of providers given that the average patient age was 34 and most cases were ascertained in the first trimester. Additionally, the rate of aneuploidy in this cohort could be impacted by the increased incidence of in vitro fertilization or other assisted reproductive technologies in multifetal pregnancies, depending on the use of pre‐implantation genetic testing of embryos and/or use of donor eggs from a donor of younger age. Data around the method of conception is not required for testing and is not routinely collected by the laboratory; therefore, this limits any assessment of the impact of those technologies on the cohort.

Another limitation of this study is that it is a retrospective review of data available to the laboratory; thus, the data is incomplete. Demographic data, including indication for testing, is derived from the test requisition forms and is therefore dependent on the accuracy of these data. Of note for multifetal pregnancies, information about the chorionicity, amnionicity, and zygosity are not routinely documented on the test requisition forms by the referring provider and are not required for testing. Thus, this study is unable to comment on the utility or performance of cfDNA screening in different types of triplet pregnancies. While efforts were made to obtain as much genetic and obstetric outcome information as possible, fewer than 10% of cases had outcome information for analysis.

## 5 Conclusion

Undoubtedly, cfDNA screening in triplet pregnancies will require additional counseling, similar to that needed for twin pregnancies. This additional counseling should cover the limited data and the higher NR rates due to more stringent requirements for fetal fraction or SNR, depending on the laboratory's particular requirements. Hopefully, most cases of triplet pregnancies are identified in the first trimester and referred to a maternal fetal medicine specialist [2] and/or a genetic counselor. However, the limited available data, including the data from this study, suggests that cfDNA screening for triplet pregnancies is a reasonable option with appropriate counseling.

## Ethics Statement

The study was conducted in accordance with the Declaration of Helsinki and approved by the Institutional Review Board at Aspire IRB under the clinical protocol SCMM‐RND‐402 (NCT04364503).

## Consent

Informed consent was not required as Aspire IRB declared that this research meets the requirements for a waiver of consent under 45 CFR 46 116(f) (2018 Requirements).

## Conflicts of Interest

All authors are current or former (SM) employees of Labcorp with the option to hold stock.

## Supporting information

Supporting Information S1

## Data Availability

The data that support the findings of this study are available from the corresponding author upon reasonable request.
