# Supplementary material for: Clinical Laboratory Experience With Prenatal cfDNA Screening in Triplet Pregnancies
Source: Prenat Diagn. 2025 May 8;45(6):705–12. doi: 10.1002/pd.6812 (PMC12137035; doi:10.1002/pd.6812)
Supplement: Supplementary file 1 — Supporting Information S1 [file PD-45-705-s001.docx]

**Supplemental Table S1: Summary of clinical details for the non-reportable cfDNA samples with available outcome information**

| **Case ID** | **cfDNA**  **Result** | **Pt age** | **GA**  **(wks)** | **Indication** | **Fetal fraction** | **Concordance** | **Outcome Notes** |
| --- | --- | --- | --- | --- | --- | --- | --- |
| 19 | QNS | 37y | 11 | Patient age | 7.20% | Other | Negative on repeat sample (Case 22: 8.23% FF, 1 week between samples). IUI pregnancy. Reduction to singleton. Delivered at term, female. Sex of other fetuses not noted. No other testing reported. |
| 34 | QNS | 34y | 10 | Patient age | 5.16% | Other | Negative on repeat sample (Case 35: 8.03% FF, 2 weeks between samples). C/S delivery at 32 wks 6 days, 1 female/2 males. No complications or other testing reported. |
| 63 | QNS | 35y | 16 | Patient age | 3.77% | Other | Negative on repeat sample (Case 65: 12.32% FF, 2 weeks between samples) . C/S delivery at 35 wks 5 days, all males. No complications or other testing reported. |
| 81 | QNS | 31y | 13 | USA | 5.51% | Other | Negative on repeat sample (Case 147: 9.53% FF, 3 weeks between samples). One fetus with cystic hygroma which resolved. Delivered 1 female/2 males (GA unspecified). No other complications or other testing reported. |
| 97 | QNS | 36y | 11 | Patient age | 6.94% | Other | Declined redraw. Delivered (GA unspecified); 3 females. No complications or other testing reported. |
| 105 | QNS | 36y | 11 | Patient age | 5.74% | Other | Declined repeat sample. Reduction with no testing on reduced triplet; CVS on triplet B was 46,XY and amniocentesis chromosomes on triplet A was 46,XY. No additional outcome information. |
| 106 | QNS | 40y | 11 | USA, Patient age | 6.48% | Other | Negative on repeat sample (Case 110: 9.56% FF, 3 weeks between samples). Two identical twins with one fraternal triplet; one identical twin had multiple USA; reduced to only the fraternal triplet (female), reported healthy. No testing on reduced triplets, noted low amniotic fluid. |
| 122 | QNS | 38y | 11 | Patient age | 5.13% | Other | No repeat sample. Reduction to singleton, then patient had an SAB. No POC testing. |
| 130 | QNS | 37y | 11 | Patient age | 5.62% | Other | Same patient as 131, redraw was also QNS. Delivered (GA unspecified); sexes not noted. No complications or other testing reported. |
| 131 | QNS | 37y | 12 | Patient age | 4.94% | Other | Same patient as 130, original was also QNS. Delivered (GA unspecified); sexes not noted. No complications or other testing reported. |
| 133 | QNS | 35y | 40 | Patient age | 5.72% | Other | No redraw. Delivered (GA unspecified); 3 females. No complications or other testing reported. |

cfDNA = prenatal cfDNA screening, Pt = Patient, GA = Gestational age, Wks = weeks, QNS = Quantity not sufficient (did not meet sample-specific fetal fraction cutoff), y = years, FF = Fetal Fraction, IUI = intrauterine insemination, C/S = Caesarean section (C-section), USA = ultrasound findings, CVS = chorionic villus samples, SAB = spontaneous abortion/miscarriage, POC = products of conception

**Supplemental Table S2: Summary of clinical details for the screen negative cases with available outcome information**

| **Case ID** | **cfDNA**  **Result** | **Pt age** | **GA**  **(wks)** | **Indication** | **Fetal fraction** | **Concordance** | **Outcome Notes** |
| --- | --- | --- | --- | --- | --- | --- | --- |
| 10 | Negative, No Y chr | 33y | 12 | None provided | 12.45% | Other | All three fetuses lost after cervical insufficiency. Fetal sexes not noted. No testing done, but patient had a subsequent singleton pregnancy that was healthy. |
| 11 | Negative, + Y chr | 27y | 19 | None provided | 22.46% | TN | Delivered at 33 wks 6 days; 2 females/1 male. No complications or other testing reported. |
| 12 | Negative, + Y chr | 30y | 10 | None provided | 16.48% | TN | Delivered at 34 wks 2 days; sexes not noted. No complications or other testing reported. |
| 13 | Negative, + Y chr | 37y | 12 | Patient age, Personal or Fam Hx | 10.18% | TN | Delivered (GA unspecified), 3 males. No complications or other testing. Fam hx of 13;14 translocation (Robertsonian); all 3 NTs normal; chr14 data unremarkable upon laboratory review. No other testing reported. |
| 14 | Negative, + Y chr | 30y | 12 | No known high risk | 11.67% | TN | Delivered at 33 wks; 1 female/2 males. No complications or other testing reported. |
| 15 | Negative, + Y chr | 31y | 12 | No known high risk | 15.81% | TN | Delivered (GA unspecified); 2 male/1 female. No complications or other testing reported. |
| 16 | Negative, No Y chr | 27y | 14 | None provided | 19.05% | Other | Delivered via C/S at 26 wks 5 days, but all three babies passed the next day. All three were female on US at 17 wks and had severe growth restriction. No other testing reported. |
| 17 | Negative, No Y chr | 36y | 12 | Patient age | 9.05% | TN | Delivered via C/S 34 wks; 3 females. Two babies went to the NICU to be monitored but were healthy. No complications or other testing reported. |
| 18 | Negative, No Y chr | 34y | 11 | Patient age | 8.88% | Other | Fetal demise of fetus B and C that the provider believes was due to chorionic entanglement. Eventually delivered preterm, 1 female. Sex of other fetuses not noted. No other testing reported. |
| 20 | Negative, + Y chr | 42y | 12 | Patient age | 13.28% | TN | Delivered at 32 wks 5 days; 2 females/1 male. No complications or other testing reported. |
| 21 | Negative, + Y chr | 28y | 13 | No known high risk | 14.38% | TN | Delivered (GA unspecified); 3 males. No complications or other testing reported. |
| 22 | Negative, + Y chr | 37y | 12 | Patient age | 8.23% | Other | Redraw of Case 19 (QNS); IUI pregnancy. Reduction to singleton. Delivered at term, female. Sex of other fetuses not noted. No other testing reported. |
| 23 | Negative, + Y chr | 19y | 19 | None provided | 8.50% | TN | Delivered at 33 wks 2 days; 2 male/1 female. All had IUGR. Triplet A had an abnormal posterior fossa, but no cause was determined, provider speculated to be due to preterm delivery. No other testing reported. |
| 24 | Negative, + Y chr | 35y | 10 | Patient age | 11.29% | TN | PPROM and chord prolapse of Triplet A, delivered preterm (GA unspecified); 2 males/1 female. No other testing reported. |
| 25 | Negative, + Y chr | 29y | 9 | None provided | 6.41% | Other | Patient had a CVS, three samples from a trichorionic/triamniotic triplet pregnancy and all are female on FISH, with no reported aneuploidy of 21/18/13. Noted another fetus that demised at 6 weeks while cfDNA was drawn at 9 weeks. Sequencing data showed Y chromosome was detected but at a lower level than the total fetal fraction and could have been contribution from the demised fetus. |
| 26 | Negative, No Y chr | 33y | 10 | No known high risk | 10.43% | Other | Reduction to singleton, but had PPROM at 15 wks 3 days, and fetus demised. No other testing reported. |
| 27 | Negative, + Y chr | 35y | 11 | Patient age | 6.57% | TN | Delivered via C/S at 32 wks 4 days. Fetal sexes not noted. No complications or other testing reported. |
| 28 | Negative, + Y chr | 38y | 22 | Patient age | 13.62% | TN | Delivered at 33 wks 6 days; 3 males. No complications or other testing reported. |
| 29 | Negative, + Y chr | 28y | 12 | None provided | 9.94% | TN | Delivered via C/S at 34 wks; 1 female/2 males. One fetus with IUGR, but otherwise no complications or other testing reported. |
| 30 | Negative, No Y chr | 28y | 11 | No known high risk | 12.77% | TN | Delivered via C/S at 28 wks 3 days; 3 females. One fetus with IUGR, but otherwise no complications or other testing reported. |
| 31 | Negative, + Y chr | 25y | 11 | None provided | 13.96% | Other | Anatomy scan showed 2 females/1 male. No anomalies noted on scan but didn't return to practice for care. |
| 32 | Negative, + Y chr | 29y | 9 | None provided | 14.65% | TN | Delivered at 31 wks 2 days; 2 females/ 1 male. No complications or other testing reported. |
| 33 | Negative, + Y chr | 35y | 9 | Patient age | 11.56% | Other | Demised fetus at 11 wks (after cfDNA). Delivered via C/S at 34 wks; 1 male, 1 female. No other testing reported. No complications reported for remaining fetuses. |
| 35 | Negative, + Y chr | 34y | 12 | Patient age | 8.03% | TN | Redraw of Case 34. C/S delivery at 32 wks 6 days, 1 female/2 males. No complications or other testing reported. |
| 36 | Negative, + Y chr | 37y | 13 | Patient age | 12.92% | TN | Delivered at 34 wks 1 day; 2 females/1 male. No complications or other testing reported. |
| 37 | Negative, + Y chr | 33y | 12 | None provided | 14.31% | TN | Reduction to singleton. Delivered at 34 wks; one male. No complications or other testing reported. Sex of other fetuses not noted. |
| 38 | Negative, + Y chr | 42y | 12 | Patient age | 10.92% | TN | Delivered via C/S at 34 wks; fetal sexes not noted. No complications or other testing reported. |
| 39 | Negative, No Y chr | 32y | 10 | None provided | 12.05% | TN | Delivered preterm (GA unspecified); 3 females. No other complications or other testing reported. |
| 40 | Negative, No Y chr | 42y | 13 | Patient age | 7.43% | TN | Delivered at 34 wks; 3 females. No complications or other testing reported. |
| 41 | Negative, + Y chr | 36y | 10 | Patient age | 8.79% | Other | Demise of one fetus. Delivered at 37 wks; 1 male/1 female. No other testing reported. |
| 42 | Negative, + Y chr | 26y | 20 | USA | 20.50% | Other | Delivered via C/S at 34 wks 5 days; 2 females/1 male. One fetus had USA (cardiac abnormality). No other testing reported. |
| 43 | Negative, + Y chr | 40y | 12 | Patient age | 6.89% | TN | Delivered via C/S at 32 wks 6 days; 1 male/2 females. No complications or other testing reported. |
| 44 | Negative, + Y chr | 30y | NP | No known high risk | 10.01% | Other | Demise of all three fetuses at 21 wks due to PPROM. Two females/1 male. No other testing reported. |
| 45 | Negative, + Y chr | 34y | 12 | None provided | 12.27% | TN | Delivered at 34 wks 4 days; 2 males/1 female. No complications or other testing reported. |
| 46 | Negative, + Y chr | 39y | 14 | Patient age | 15.71% | TN | Delivered at 34 weeks; fetal sexes not noted. No complications or other testing reported. |
| 47 | Negative, + Y chr | 34y | 12 | Patient age | 8.01% | TN | Delivered (GA unspecified); 3 males. No complications or other testing reported. |
| 48 | Negative, No Y chr | 36y | 15 | Patient age | 14.98% | TN | Delivered at 32 wks 1 day; 3 females. No complications or other testing reported. |
| 50 | Negative, No Y chr | 29y | 12 | None provided | 9.99% | TN | Delivered at 32 wks 4 days; 3 females. Patient had severe preeclampsia and all three fetuses had IUGR. No other testing reported. |
| 51 | Negative, + Y chr | 35y | 12 | Patient age | 13.17% | TN | Delivered at 33 wks 4 days; 1 female/2 males. No complications or other testing reported. |
| 52 | Negative, No Y chr | 27y | 17 | USA | 10.35% | TN | Delivered (GA unspecified); 3 females. No complications or other testing reported. |
| 53 | Negative, No Y chr | 36y | 11 | Patient age | 8.00% | TN | Delivered at 34 wks 1 day; 3 females. Triplet B with IUGR. No other complications or other testing reported. |
| 54 | Negative, No Y chr | 23y | 19 | Personal or fam hx | 14.08% | TN | Delivered at 32 weeks; 3 females. No complications or other testing reported. |
| 55 | Negative, No Y chr | 36y | 17 | Patient age | 19.98% | TN | Delivered at 35 wks 1 day; 3 females. Patient had postpartum hemorrhage but recovered well. Placental testing (unspecified) came back normal. No other complications or other testing reported. |
| 56 | Negative, + Y chr | 31y | 13 | None provided | 15.37% | TN | Delivered preterm at 28 wks 4 days; 2 female/1 male. No complications or other testing reported. |
| 57 | Negative, + Y chr | 34y | 10 | None provided | 13.06% | Other | Last note from OB said patient was carrying triplets normally. No other notes regarding further testing or delivery. |
| 58 | Negative, No Y chr | 27y | 14 | Personal or Fam Hx | 8.69% | LN | Delivered at 33 wks; fetal sexes not noted. Delivered due to growth restriction, echo on fetuses was normal. No other complications or other testing reported. |
| 59 | Negative, + Y chr | 33y | 12 | None provided | 11.28% | TN | Delivered at 31 wks 4 days; fetal sexes not noted. Severe preeclampsia, may have had an abruption. No other complications or other testing reported. |
| 60 | Negative, + Y chr | 34y | 26 | Patient age | 13.87% | TN | Delivered at 38 weeks, 1 day; fetal sexes not noted. No complications or other testing reported. |
| 61 | Negative, No Y chr | 39y | 12 | Patient age | 7.53% | Other | Delivered via C/S at 29 wks 4 days, Baby B had demised. Fetal sexes not noted. No other complications or other testing reported. |
| 62 | Negative, + Y chr | 30y | 10 | None provided | 15.09% | TN | Delivered at 33 wks 2 days; fetal sexes no noted. No complications or other testing reported. |
| 64 | Negative, + Y chr | 41y | 10 | Patient age | 11.82% | Other | Reduction of one fetus, as they were experiencing IUGR. Delivered via C/S at 35 weeks; 2 females. Noted a fourth fetus that demised before cfDNA test. No sex noted for reduced fetus. No other complications or other testing reported. |
| 65 | Negative, + Y chr | 35y | 18 | Patient age | 12.32% | TN | Redraw of Case 63 (QNS). C/S delivery at 35 wks 5 days; 3 males. No complications or other testing reported. |
| 66 | Negative, + Y chr | 37y | 11 | Patient age | 19.23% | TN | Delivered via C/S at 36 wks; 1 female/2 males. No complications or other testing reported. |
| 67 | Negative, + Y chr | 23y | 12 | None provided | 10.04% | TN | Delivered via C/s at 27 wks; 1 male/2 female. No other complications or testing reported. |
| 68 | Negative, Opted out of fetal sex | 36y | 13 | Patient age | 10.87% | Other | Reduction of one fetus at 18 wks due to neural tube defect. Delivered at 34 wks; 1 male/1 female. No other complications or testing reported. |
| 69 | Negative, + Y chr | 33y | 18 | None provided | 10.16% | TN | Delivered at 34 wks; fetal sexes not noted. No complications or other testing reported. |
| 70 | Negative, No Y chr | 31y | 11 | None provided | 10.14% | Other | Fetal demise of all three fetuses at 20 wks 2 days. Array on amniotic fluid showed 850 kb deletion on 15q11.2->q11.2 on Triplet C. Not detectable by standard cfDNA, did not order microdeletions. VSD and cystic hygroma on triplet C. |
| 71 | Negative, + Y chr | 30y | 12 | None provided | 7.24% | TN | Delivered (GA unspecified); 2 males/1 female. No complications or other testing reported. |
| 72 | Negative, + Y chr | 30y | 12 | None provided | 9.32% | TN | Delivered at 35 wks; 2 males/1 female. No complications or other testing reported. |
| 73 | Negative, No Y chr | 28y | 15 | No known high risk | 12.58% | TN | Delivered at 34 wks 4 days; 3 females. No complications or other testing reported. |
| 74 | Negative, + Y chr | 25y | 12 | None provided | 12.25% | TN | Delivered (GA unspecified); 2 males/1 female. No complications or other testing reported. |
| 75 | Negative, + Y chr | 31y | 9 | No known high risk | 6.89% | TN | Normal male for all three fetuses on CVS karyotype and array. No pregnancy outcome information available. |
| 76 | Negative, + Y chr | 37y | 10 | Patient age | 10.68% | TN | Delivered (GA unspecified); 1 male/2 females. No complications or other testing reported. |
| 77 | Negative, + Y chr | 37y | 11 | Patient age | 7.18% | TN | Delivered (GA unspecified); 2 males/1 female. No complications or other testing reported. |
| 78 | Negative, No Y chr | 37y | 22 | Patient age | 8.97% | TN | Delivered (GA unspecified); fetal sexes not noted. No complications or other testing reported. |
| 79 | Negative, + Y chr | 33y | 12 | No known high risk | 12.57% | TN | Delivered via C/S at 33 wks; 1 male/2 females. Placental abruption. No complications or other testing reported. |
| 80 | Negative, Opted out of fetal sex | 35y | 11 | Patient age | 13.71% | TN | Delivered at 32 wks; 2 females/1 male at 32 wks. No complications or other testing reported. |
| 82 | Negative, + Y chr | 33y | 15 | No known high risk | 12.59% | TN | Delivered (GA unspecified); 2 males/1 female. No complications or other testing reported. |
| 83 | Negative, + Y chr | 34y | 12 | No known high risk | 14.45% | TN | Delivered (GA unspecified); 2 males/1 female. No complications or other testing reported. |
| 84 | Negative, + Y chr | 28y | 14 | None provided | 11.36% | TN | Delivered at 27 weeks 6 days; 2 females/1 male. No complications or other testing reported. |
| 85 | Negative, + Y chr | 25y | 9 | No known high risk | 15.85% | Other | Delivered two babies (GA unspecified). Noted at least one boy, but provider could not see further documentation as to pregnancy outcome. |
| 86 | Negative, + Y chr | 32y | 12 | No known high risk | 18.79% | TN | Delivered (GA unspecified; 2 females/1 male. No complications or other testing reported. |
| 87 | Negative, No Y chr | 36y | 13 | Patient age | 7.66% | Other | Delivered (GA unspecified but noted as preterm); 3 females. One baby had acrania. No other complications or other testing reported. |
| 88 | Negative, + Y chr | 36y | 12 | Patient age | 12.16% | TN | Delivered (GA unspecified); 2 females/1 male. No complications or other testing reported. |
| 89 | Negative, + Y chr | 45y | 12 | Patient age | 10.29% | TN | Delivered (GA unspecified); 3 males. Babies went to NICU, but no concern for chromosome abnormalities. |
| 90 | Negative, + Y chr | 24y | 17 | Patient age | 9.62% | TN | Delivered at 28 wks 1 day; 2 females/1 male. Presented with membranes ruptured, but no other complications or other testing reported. |
| 91 | Negative, No Y chr | 31y | 26 | USA | 20.38% | TN | Delivered (GA unspecified); 3 females. No complications or other testing reported. |
| 92 | Negative, + Y chr | 33y | 13 | Personal or fam hx | 8.59% | TN | Delivered (GA unspecified); 2 females/1 male. No complications or other testing reported. |
| 93 | Negative, No Y chr | 35y | 12 | USA, Patient age | 9.69% | TN | Delivered (GA unspecified, but noted preterm); 3 females. Went to NICU due to premature delivery but did well. No other complications or other testing reported. |
| 94 | Negative, + Y chr | 36y | 12 | USA, Patient age | 6.21% | TN | Delivered (GA unspecified); 2 females/1 male. No complications or other testing reported. |
| 95 | Negative, No Y chr | 35y | 13 | Patient age | 10.30% | TN | Delivered (GA unspecified); 3 females. No complications or other testing reported. |
| 96 | Negative, No Y chr | 37y | 10 | Patient age | 15.34% | Other | Reduction to singleton. Delivered (GA unspecified); 1 female. Did amnio microarray on singleton, was 46,XX. No other complications reported. |
| 98 | Negative, + Y chr | 25y | 18 | No known high risk | 14.90% | TN | Delivered (GA unspecified); 3 males. No complications or other testing reported. |
| 99 | Negative, + Y chr | 38y | 12 | Patient age | 12.48% | TN | Delivered (GA unspecified); 3 males. No complications or other testing reported. |
| 100 | Negative, No Y chr | 40y | 12 | Patient age | 12.68% | TN | Delivered (GA unspecified); 3 females. No complications or other testing reported. |
| 102 | Negative, + Y chr | 36y | 10 | Patient age | 9.50% | TN | Delivered (GA unspecified); 2 females/1 male. No complications or other testing reported. |
| 103 | Negative, Opted out of fetal sex | 38y | 10 | Patient age | 11.34% | TN | Delivered (GA unspecified); fetal sexes not noted No complications or other testing reported. |
| 104 | Negative, + Y chr | 26y | 18 | No known high risk | 14.19% | TN | Delivered (GA unspecified); 2 males/1 female. No complications or other testing reported. |
| 107 | Negative, + Y chr | 35y | 13 | Patient age | 15.44% | TN | Delivered (GA unspecified); 2 males/1 female. No complications or other testing reported. |
| 108 | Negative, + Y chr | 36y | 11 | Patient age | 13.00% | TN | Delivered (GA unspecified); 2 females/1 male. No complications or other testing reported. |
| 109 | Negative, + Y chr | 38y | 18 | Patient age | 17.31% | TN | Delivered at 29 wks; 1 female/2 males. No complications or other testing reported. |
| 110 | Negative, No Y chr | 40y | 14 | Patient age | 9.56% | Other | Redraw of Case 106. Two identical twins with one fraternal triplet; one identical twin had multiple USA; reduced to only the fraternal triplet (female), reported healthy. No testing on reduced triplets, noted low amniotic fluid. |
| 111 | Negative, + Y chr | 37y | 12 | Patient age | 8.82% | TN | Delivered (GA unspecified); 1 female/2 males. No complications or other testing reported. |
| 112 | Negative, + Y chr | 37y | 12 | Patient age | 20.62% | TN | Delivered (GA unspecified); 1 female/2 males. No complications or other testing reported. |
| 113 | Negative, No Y chr | 46y | 11 | Patient age | 9.25% | TN | Delivered (GA unspecified); 3 females. No complications or other testing reported. |
| 114 | Negative, + Y chr | 37y | 20 | Patient age | 13.94% | TN | Delivered (GA unspecified); 2 females/1 male. No complications or other testing reported. |
| 115 | Negative, + Y chr | 39y | 11 | Patient age | 17.03% | TN | Delivered at 29 weeks; 2 females/1 male. No complications or other testing reported |
| 116 | Negative, + Y chr | 41y | 10 | USA, Patient age | 7.90% | TN | Delivered (GA unspecified); 3 males. One triplet had enlarged NT 3.8, but that resolved. No other complications or other testing reported. |
| 117 | Negative, No Y chr | 42y | 12 | Patient age | 16.88% | Other | One triplet demised at ~24 wks, no POC testing. Delivered (GA unspecified); 2 females. No other complications or other testing reported. |
| 118 | Negative, + Y chr | 44y | 12 | Patient age | 9.02% | TN | Delivered (GA unspecified); fetal sexes not noted No complications or other testing reported. |
| 120 | Negative, + Y chr | 34y | 20 | USA | 7.27% | TN | Delivered at 34 wks 3 days; 2 females/1 male. One triplet had a CPC, but it resolved. No complications or other testing reported. |
| 121 | Negative, + Y chr | 37y | 14 | Patient age | 9.58% | TN | Delivered (GA unspecified); fetal sexes not noted. No complications or other testing reported. |
| 123 | Negative, No Y chr | 36y | 12 | Patient age | 12.50% | Other | Reduction to singleton, no diagnostic testing. Delivered (GA unspecified), female. No complications or other testing reported. |
| 124 | Negative, Opted out of fetal sex | 24y | 24 | USA | 16.14% | TN | Delivered (GA unspecified); fetal sexes not noted. No complications or other testing reported. |
| 125 | Negative, + Y chr | 37y | 9 | Patient age | 8.43% | TN | Delivered (GA unspecified); 1 female/2 males. No complications or other testing reported. |
| 126 | Negative, + Y chr | 24y | 18 | No known high risk | 8.92% | Other | Pregnancy loss of all three fetuses, no POC testing. |
| 127 | Negative, No Y chr | 35y | 11 | Patient age, Personal or fam hx | 10.36% | TN | Delivered (GA unspecified); 3 females. No complications or other testing reported. |
| 128 | Negative, + Y chr | 38y | 13 | Patient age | 12.64% | TN | Delivered (GA unspecified); 2 females/1 male. No complications or other testing reported. |
| 129 | Negative, + Y chr | 29y | 12 | USA | 8.99% | Other | Both patient and partner carriers of Meckel-Gruber syndrome. Reduction to singleton, as two fetuses were showing USA consistent with Meckel-Gruber Syndrome. No fetal testing. Delivered (GA unspecified), male. No other complications or other testing. |
| 132 | Negative, Opted out of fetal sex | 35y | 13 | Patient age | 12.56% | TN | Delivered (GA unspecified); 2 females/1 male. No complications or other testing reported. |
| 134 | Negative, + Y chr | 26y | 16 | None provided | 7.51% | TN | Delivered (GA unspecified); 1 female/2 males. No complications or other testing reported. |
| 135 | Negative, + Y chr | 39y | 19 | Patient age | 13.73% | TN | Delivered (GA unspecified); 2 females/1 male. No complications or other testing reported. |
| 136 | Negative, + Y chr | 31y | 12 | None provided | 9.50% | Other | Preterm labor with demise of all three fetuses at 21 wks 3 days. At least two were male, but sex of third fetus unclear. No testing before or after the demise, no clear cause of early labor. |
| 137 | Negative, Opted out of fetal sex | 36y | 10 | Patient age | 10.35% | Other | Reduction to singleton. No reported testing on any of the fetuses no available details regarding delivery. |
| 138 | Negative, + Y chr | 28y | 11 | None provided | 6.79% | TN | Delivered (GA unspecified); 2 females/1 male. No complications or other testing reported. |
| 139 | Negative, No Y chr | 29y | 17 | None provided | 21.64% | Other | Monochorionic triplets; early pregnancy loss at 22 wks, all female but no POC testing. |
| 140 | Negative, + Y chr | 32y | 10 | None provided | 9.42% | Other | Reduction to twins due to multiple defects. No POC testing, but noted all three were boys. Delivered (GA unspecified); 2 males. No other complications or other testing reported. |
| 141 | Negative, + Y chr | 22y | 10 | None provided | 9.24% | Other | Stillbirth at 18 wks of one triplet due to PPROM, then emergency C/S at 23 wks with neonatal demise of other two. No additional testing reported. |
| 142 | Negative, + Y chr | 31y | 12 | None provided | 16.61% | TN | Delivered (GA unspecified); 2 females/1 male. No complications or other testing reported. |
| 144 | Negative, + Y chr | 35y | 25 | Patient age | 29.80% | TN | Delivered (GA unspecified); 2 females/1 male. No complications or other testing reported. |
| 145 | Negative, No Y chr | 39y | 18 | Patient age | 18.01% | TN | Delivered (GA unspecified); 3 females. No complications or other testing reported. |
| 146 | Negative, + Y chr | 34y | 12 | Personal or fam hx | 5.50% | TN | Delivered (GA unspecified); 1 female/2 males. No complications or other testing reported. |
| 147 | Negative, + Y chr | 31y | 16 | USA | 9.53% | TN | Redraw of Case 81. One fetus with cystic hygroma which resolved. Delivered 1 female/2 males (GA unspecified). No other complications or other testing reported. |

cfDNA = prenatal cfDNA screening, Pt = Patient, GA = Gestational age, Wks = weeks, No Y chr = No Y chromosome detected in sample, y = years, TN = true negative, + Y Chr = Y chromosome detected in sample, Fam hx = family history, GA = gestational age, NT = nuchal translucency, chr = chromosome, C/S = Caesarean section (C-section, US = Ultrasound, NICU = Neonatal intensive care unit, IUI = intrauterine insemination, IUGR = intrauterine growth restriction, PPROM = preterm premature rupture of membranes, CVS = chorionic villus sampling, NP = not provided; OB = obstetrician; kb = kilobase, POC = products of conception**,** CPC = choroid plexus cyst

**Supplemental Table S3.** Available fetal sex outcomes for study cohort.

| **ID** | **cfDNA**  **Result** | **Pt age** | **GA**  **(wks)** | **Indication** | **Fetal fraction** | **Concordance** | **Outcome Notes** |
| --- | --- | --- | --- | --- | --- | --- | --- |
| 11 | Negative, + Y chr | 27y | 19 | None provided | 22.46% | TN | Delivered at 33 wks 6 days; 2 females/1 male. No complications or other testing reported. |
| 13 | Negative, + Y chr | 37y | 12 | Patient age, Personal or Fam Hx | 10.18% | TN | Delivered (GA unspecified), 3 males. No complications or other testing. Fam hx of 13;14 translocation (Robertsonian); all 3 NTs normal; chr14 data unremarkable upon laboratory review. No other testing reported. |
| 14 | Negative, + Y chr | 30y | 12 | No known high risk | 11.67% | TN | Delivered at 33 wks; 1 female/2 males. No complications or other testing reported. |
| 15 | Negative, + Y chr | 31y | 12 | No known high risk | 15.81% | TN | Delivered (GA unspecified); 2 male/1 female. No complications or other testing reported. |
| 16 | Negative, No Y chr | 27y | 14 | None provided | 19.05% | Other | Delivered via C/S at 26 wks 5 days, but all three babies passed the next day. All three were female on US at 17 wks and had severe growth restriction. No other testing reported. |
| 17 | Negative, No Y chr | 36y | 12 | Patient age | 9.05% | TN | Delivered via C/S 34 wks; 3 females. Two babies went to the NICU to be monitored but were healthy. No complications or other testing reported. |
| 20 | Negative, + Y chr | 42y | 12 | Patient age | 13.28% | TN | Delivered at 32 wks 5 days; 2 females/1 male. No complications or other testing reported. |
| 21 | Negative, + Y chr | 28y | 13 | No known high risk | 14.38% | TN | Delivered (GA unspecified); 3 males. No complications or other testing reported. |
| 23 | Negative, + Y chr | 19y | 19 | None provided | 8.50% | TN | Delivered at 33 wks 2 days; 2 male/1 female. All had IUGR. Triplet A had an abnormal posterior fossa, but no cause was determined, provider speculated to be due to preterm delivery. No other testing reported. |
| 24 | Negative, + Y chr | 35y | 10 | Patient age | 11.29% | TN | PPROM and chord prolapse of Triplet A, delivered preterm (GA unspecified); 2 males/1 female. No other testing reported. |
| 25 | Negative, + Y chr | 29y | 9 | None provided | 6.41% | Other | Patient had a CVS, three samples from a trichorionic/triamniotic triplet pregnancy and all are female on FISH, with no reported aneuploidy of 21/18/13. Noted another fetus that demised at 6 weeks while cfDNA was drawn at 9 weeks. Sequencing data showed Y chromosome was detected but at a lower level than the total fetal fraction and could have been contribution from the demised fetus. |
| 28 | Negative, + Y chr | 38y | 22 | Patient age | 13.62% | TN | Delivered at 33 wks 6 days; 3 males. No complications or other testing reported. |
| 29 | Negative, + Y chr | 28y | 12 | None provided | 9.94% | TN | Delivered via C/S at 34 wks; 1 female/2 males. One fetus with IUGR, but otherwise no complications or other testing reported. |
| 30 | Negative, No Y chr | 28y | 11 | No known high risk | 12.77% | TN | Delivered via C/S at 28 wks 3 days; 3 females. One fetus with IUGR, but otherwise no complications or other testing reported. |
| 31 | Negative, + Y chr | 25y | 11 | None provided | 13.96% | Other | Anatomy scan showed 2 females/1 male. No anomalies noted on scan but didn't return to practice for care. |
| 32 | Negative, + Y chr | 29y | 9 | None provided | 14.65% | TN | Delivered at 31 wks 2 days; 2 females/ 1 male. No complications or other testing reported. |
| 35 | Negative, + Y chr | 34y | 12 | Patient age | 8.03% | TN | Redraw of Case 34. C/S delivery at 32 wks 6 days, 1 female/2 males. No complications or other testing reported. |
| 36 | Negative, + Y chr | 37y | 13 | Patient age | 12.92% | TN | Delivered at 34 wks 1 day; 2 females/1 male. No complications or other testing reported. |
| 39 | Negative, No Y chr | 32y | 10 | None provided | 12.05% | TN | Delivered preterm (GA unspecified); 3 females. No other complications or other testing reported. |
| 40 | Negative, No Y chr | 42y | 13 | Patient age | 7.43% | TN | Delivered at 34 wks; 3 females. No complications or other testing reported. |
| 42 | Negative, + Y chr | 26y | 20 | USA | 20.50% | Other | Delivered via C/S at 34 wks 5 days; 2 female/1 male. One fetus had USA (cardiac abnormality). No other testing reported. |
| 43 | Negative, + Y chr | 40y | 12 | Patient age | 6.89% | TN | Delivered via C/S at 32 wks 6 days; 1 male/2 females. No complications or other testing reported. |
| 44 | Negative, + Y chr | 30y | NP | No known high risk | 10.01% | Other | Demise of all three fetuses at 21 wks due to PPROM. Two females/1 male. No other testing reported. |
| 45 | Negative, + Y chr | 34y | 12 | None provided | 12.27% | TN | Delivered at 34 wks 4 days; 2 males/1 female. No complications or other testing reported. |
| 47 | Negative, + Y chr | 34y | 12 | Patient age | 8.01% | TN | Delivered (GA unspecified); 3 males. No complications or other testing reported. |
| 48 | Negative, No Y chr | 36y | 15 | Patient age | 14.98% | TN | Delivered at 32 wks 1 day; 3 females. No complications or other testing reported. |
| 50 | Negative, No Y chr | 29y | 12 | None provided | 9.99% | TN | Delivered at 32 wks 4 days; 3 females. Patient had severe preeclampsia and all three fetuses had IUGR. No other testing reported. |
| 51 | Negative, + Y chr | 35y | 12 | Patient age | 13.17% | TN | Delivered at 33 wks 4 days; 1 female/2 males. No complications or other testing reported. |
| 52 | Negative, No Y chr | 27y | 17 | USA | 10.35% | TN | Delivered (GA unspecified); 3 females. No complications or other testing reported. |
| 53 | Negative, No Y chr | 36y | 11 | Patient age | 8.00% | TN | Delivered at 34 wks 1 day; 3 females. Triplet B with IUGR. No other complications or other testing reported. |
| 54 | Negative, No Y chr | 23y | 19 | Personal or fam hx | 14.08% | TN | Delivered at 32 weeks; 3 females. No complications or other testing reported. |
| 55 | Negative, No Y chr | 36y | 17 | Patient age | 19.98% | TN | Delivered at 35 wks 1 day; 3 females. Patient had postpartum hemorrhage but recovered well. Placental testing (unspecified) came back normal. No other complications or other testing reported. |
| 56 | Negative, + Y chr | 31y | 13 | None provided | 15.37% | TN | Delivered preterm at 28 wks 4 days; 2 female/1 male. No complications or other testing reported. |
| 65 | Negative, + Y chr | 35y | 18 | Patient age | 12.32% | TN | Redraw of Case 63 (QNS). C/S delivery at 35 wks 5 days; 3 males. No complications or other testing reported. |
| 66 | Negative, + Y chr | 37y | 11 | Patient age | 19.23% | TN | Delivered via C/S at 36 wks; 1 female/2 males. No complications or other testing reported. |
| 67 | Negative, + Y chr | 23y | 12 | None provided | 10.04% | TN | Delivered via C/s at 27 wks; 1 male/2 female. No other complications or testing reported. |
| 71 | Negative, + Y chr | 30y | 12 | None provided | 7.24% | TN | Delivered (GA unspecified); 2 males/1 female. No complications or other testing reported. |
| 72 | Negative, + Y chr | 30y | 12 | None provided | 9.32% | TN | Delivered at 35 wks; 2 males/1 female. No complications or other testing reported. |
| 73 | Negative, No Y chr | 28y | 15 | No known high risk | 12.58% | TN | Delivered at 34 wks 4 days; 3 females. No complications or other testing reported. |
| 74 | Negative, + Y chr | 25y | 12 | None provided | 12.25% | TN | Delivered (GA unspecified); 2 males/1 female. No complications or other testing reported. |
| 75 | Negative, + Y chr | 31y | 9 | No known high risk | 6.89% | TN | Normal male for all three fetuses on CVS karyotype and array. No pregnancy outcome information available. |
| 76 | Negative, + Y chr | 37y | 10 | Patient age | 10.68% | TN | Delivered (GA unspecified); 1 male/2 females. No complications or other testing reported. |
| 77 | Negative, + Y chr | 37y | 11 | Patient age | 7.18% | TN | Delivered (GA unspecified); 2 males/1 female. No complications or other testing reported. |
| 79 | Negative, + Y chr | 33y | 12 | No known high risk | 12.57% | TN | Delivered via C/S at 33 wks; 1 male/2 females. Placental abruption. No complications or other testing reported. |
| 80 | Negative, Opted out of fetal sex | 35y | 11 | Patient age | 13.71% | TN | Delivered at 32 wks; 2 females/1 male at 32 wks. No complications or other testing reported. |
| 82 | Negative, + Y chr | 33y | 15 | No known high risk | 12.59% | TN | Delivered (GA unspecified); 2 males/1 female. No complications or other testing reported. |
| 83 | Negative, + Y chr | 34y | 12 | No known high risk | 14.45% | TN | Delivered (GA unspecified); 2 males/1 female. No complications or other testing reported. |
| 84 | Negative, + Y chr | 28y | 14 | None provided | 11.36% | TN | Delivered at 27 weeks 6 days; 2 females/1 male. No complications or other testing reported. |
| 86 | Negative, + Y chr | 32y | 12 | No known high risk | 18.79% | TN | Delivered (GA unspecified; 2 females/1 male. No complications or other testing reported. |
| 87 | Negative, No Y chr | 36y | 13 | Patient age | 7.66% | Other | Delivered (GA unspecified but noted as preterm); 3 females. One baby had acrania. No other complications or other testing reported. |
| 88 | Negative, + Y chr | 36y | 12 | Patient age | 12.16% | TN | Delivered (GA unspecified); 2 females/1 male. No complications or other testing reported. |
| 89 | Negative, + Y chr | 45y | 12 | Patient age | 10.29% | TN | Delivered (GA unspecified); 3 males. Babies went to NICU, but no concern for chromosome abnormalities. |
| 90 | Negative, + Y chr | 24y | 17 | Patient age | 9.62% | TN | Delivered at 28 wks 1 day; 2 females/1 male. Presented with membranes ruptured, but no other complications or other testing reported. |
| 91 | Negative, No Y chr | 31y | 26 | USA | 20.38% | TN | Delivered (GA unspecified); 3 females. No complications or other testing reported. |
| 92 | Negative, + Y chr | 33y | 13 | Personal or fam hx | 8.59% | TN | Delivered (GA unspecified); 2 females/1 male. No complications or other testing reported. |
| 93 | Negative, No Y chr | 35y | 12 | USA, Patient age | 9.69% | TN | Delivered (GA unspecified, but noted preterm); 3 females. Went to NICU due to premature delivery but did well. No other complications or other testing reported. |
| 94 | Negative, + Y chr | 36y | 12 | USA, Patient age | 6.21% | TN | Delivered (GA unspecified); 2 females/1 male. No complications or other testing reported. |
| 95 | Negative, No Y chr | 35y | 13 | Patient age | 10.30% | TN | Delivered (GA unspecified); 3 females. No complications or other testing reported. |
| 98 | Negative, + Y chr | 25y | 18 | No known high risk | 14.90% | TN | Delivered (GA unspecified); 3 males. No complications or other testing reported. |
| 99 | Negative, + Y chr | 38y | 12 | Patient age | 12.48% | TN | Delivered (GA unspecified); 3 males. No complications or other testing reported. |
| 100 | Negative, No Y chr | 40y | 12 | Patient age | 12.68% | TN | Delivered (GA unspecified); 3 females. No complications or other testing reported. |
| 102 | Negative, + Y chr | 36y | 10 | Patient age | 9.50% | TN | Delivered (GA unspecified); 2 females/1 male. No complications or other testing reported. |
| 104 | Negative, + Y chr | 26y | 18 | No known high risk | 14.19% | TN | Delivered (GA unspecified); 2 males/1 female. No complications or other testing reported. |
| 107 | Negative, + Y chr | 35y | 13 | Patient age | 15.44% | TN | Delivered (GA unspecified); 2 males/1 female. No complications or other testing reported. |
| 108 | Negative, + Y chr | 36y | 11 | Patient age | 13.00% | TN | Delivered (GA unspecified); 2 females/1 male. No complications or other testing reported. |
| 109 | Negative, + Y chr | 38y | 18 | Patient age | 17.31% | TN | Delivered at 29 wks; 1 female/2 males. No complications or other testing reported. |
| 111 | Negative, + Y chr | 37y | 12 | Patient age | 8.82% | TN | Delivered (GA unspecified); 1 female/2 males. No complications or other testing reported. |
| 112 | Negative, + Y chr | 37y | 12 | Patient age | 20.62% | TN | Delivered (GA unspecified); 1 female/2 males. No complications or other testing reported. |
| 113 | Negative, No Y chr | 46y | 11 | Patient age | 9.25% | TN | Delivered (GA unspecified); 3 females. No complications or other testing reported. |
| 114 | Negative, + Y chr | 37y | 20 | Patient age | 13.94% | TN | Delivered (GA unspecified); 2 females/1 male. No complications or other testing reported. |
| 115 | Negative, + Y chr | 39y | 11 | Patient age | 17.03% | TN | Delivered at 29 weeks; 2 females/1 male. No complications or other testing reported |
| 116 | Negative, + Y chr | 41y | 10 | USA, Patient age | 7.90% | TN | Delivered (GA unspecified); 3 males. One triplet had enlarged NT 3.8, but that resolved. No other complications or other testing reported. |
| 120 | Negative, + Y chr | 34y | 20 | USA | 7.27% | TN | Delivered at 34 wks 3 days; 2 females/1 male. One triplet had a CPC, but it resolved. No complications or other testing reported. |
| 125 | Negative, + Y chr | 37y | 9 | Patient age | 8.43% | TN | Delivered (GA unspecified); 1 female/2 males. No complications or other testing reported. |
| 127 | Negative, No Y chr | 35y | 11 | Patient age, Personal or fam hx | 10.36% | TN | Delivered (GA unspecified); 3 females. No complications or other testing reported. |
| 128 | Negative, + Y chr | 38y | 13 | Patient age | 12.64% | TN | Delivered (GA unspecified); 2 females/1 male. No complications or other testing reported. |
| 132 | Negative, Opted out of fetal sex | 35y | 13 | Patient age | 12.56% | TN | Delivered (GA unspecified); 2 females/1 male. No complications or other testing reported. |
| 134 | Negative, + Y chr | 26y | 16 | None provided | 7.51% | TN | Delivered (GA unspecified); 1 female/2 males. No complications or other testing reported. |
| 135 | Negative, + Y chr | 39y | 19 | Patient age | 13.73% | TN | Delivered (GA unspecified); 2 females/1 male. No complications or other testing reported. |
| 138 | Negative, + Y chr | 28y | 11 | None provided | 6.79% | TN | Delivered (GA unspecified); 2 females/1 male. No complications or other testing reported. |
| 139 | Negative, No Y chr | 29y | 17 | None provided | 21.64% | Other | Monochorionic triplets; early pregnancy loss at 22 wks, all female but no POC testing. |
| 140 | Negative, + Y chr | 32y | 10 | None provided | 9.42% | Other | Reduction to twins due to multiple defects. No POC testing, but noted all three were boys. Delivered (GA unspecified); 2 males. No other complications or other testing reported. |
| 142 | Negative, + Y chr | 31y | 12 | None provided | 16.61% | TN | Delivered (GA unspecified); 2 females/1 male. No complications or other testing reported. |
| 144 | Negative, + Y chr | 35y | 25 | Patient age | 29.80% | TN | Delivered (GA unspecified); 2 females/1 male. No complications or other testing reported. |
| 145 | Negative, No Y chr | 39y | 18 | Patient age | 18.01% | TN | Delivered (GA unspecified); 3 females. No complications or other testing reported. |
| 146 | Negative, + Y chr | 34y | 12 | Personal or fam hx | 5.50% | TN | Delivered (GA unspecified); 1 female/2 males. No complications or other testing reported. |
| 147 | Negative, + Y chr | 31y | 16 | USA | 9.53% | TN | Redraw of Case 81. One fetus with cystic hygroma which resolved. Delivered 1 female/2 males (GA unspecified). No other complications or other testing reported. |

**Supplemental Table S4**

|  | N (wt) | Med  wt | Avg wt | IQR wt | Med FF | Avg FF | IQR FF | Med GA | Avg GA | IQR GA | Med MA | Avg MA | IQR MA |
| --- | --- | --- | --- | --- | --- | --- | --- | --- | --- | --- | --- | --- | --- |
| QNS/TNR cohort | 250 | 185 | 192.156 | 60 | 0.070 | 0.074 | 0.038 | 12 | 12.923 | 3 | 35 | 34.31 | 7 |
| Report cohort | 1152 | 155 | 162.725 | 45 | 0.122 | 0.127 | 0.053 | 12 | 13.274 | 3 | 35 | 34.04 | 6 |

N=sample size, Med wt = Median weight, Avg wt = Average weight, IQR wt = Interquartile range for weight, Med FF = Median fetal fraction, Avg FF = Average fetal fraction, IQR FF = Interquartile range for fetal fraction, Med GA = Median gestational age, Avg GA = Average gestational age, IQR GA = Interquartile range for gestational age, Med MA = Median maternal age, Avg MA = Average maternal age, IQR MA = Interquartile range for maternal age, QNS = Quantity not sufficient, TNR = technical non-reportable
